# Feelings of loneliness and mental health needs and services utilization among Chinese residents during the COVID-19 epidemic

**DOI:** 10.1186/s12992-021-00704-5

**Published:** 2021-04-26

**Authors:** Li Bao, Wen-Tian Li, Bao-Liang Zhong

**Affiliations:** 1 Department of Psychiatry, Wuhan Mental Health Center, No. 89, Gongnongbing Road, Hubei Province 430012 Wuhan, China; 2 Department of Psychiatry, Affiliated Wuhan Mental Health Center, Tongji Medical College of Huazhong, University of Science & Technology, 430012 Wuhan, Hubei Province China

**Keywords:** Loneliness, Mental health needs, Mental health services, COVID-19

## Abstract

**Background:**

Due to the implementation of social distancing and quarantine measures, loneliness has been a major public health concern during the COVID-19 pandemic. However, few studies have examined loneliness in Chinese residents during the COVID-19 epidemic, as well as its associations with mental health needs and services utilization.

**Methods:**

The present study was a cross-sectional survey during the COVID-19 outbreak in China. A total of 7741 adults were invited and completed an online self-administered questionnaire. The Chinese 12-item General Health Questionnaire was used to screen for common mental health problems, loneliness was measured with a single-item self-report question (“How often do you feel lonely in recent days?”), and two standardized questions were used to assess perceived needs for and use of mental health services.

**Results:**

In total, 24.2 % of the participants felt lonely in recent days. Age of 16–29 years (OR = 1.36, *P* = 0.020), marital status of never-married (OR = 1.47, *P* < 0.001), marital status of “others” (re-married, co-habiting, separated, divorced, and widowed) (OR = 1.72, *P* < 0.001), having infected family members or close relatives (OR = 1.64, *P* = 0.026), and having infected colleagues, friends, or classmates (OR = 1.62, *P* < 0.001) were significant correlates of loneliness. Rates of mental health needs (17.4 % vs. 4.9 %, *P* < 0.001) and services utilization (2.7 % vs. 1.0 %, *P* < 0.001) were significantly higher in lonely than not lonely participants. After adjusting for socio-demographic and epidemic characteristics and common mental health problems, loneliness was still significantly associated with mental health needs (OR = 2.50, *P* < 0.001) and services utilization (OR = 1.62, *P* = 0.020).

**Conclusions:**

Feelings of loneliness are prevalent among Chinese residents affected by the COVID-19 epidemic and the presence of loneliness is associated with high levels of mental health needs and greater services utilization. Effective measures aiming at preventing or reducing loneliness are potentially beneficial for the mental wellbeing of COVID-19-affected population and reducing the use of the limited mental health service resources during the COVID-19 pandemic.

## Introduction

The ongoing COVID-19 pandemic has posed unprecedented challenges to the mental health of people all around the world [1]. To reduce the spread of COVID-19, various containment strategies were adopted, including lockdowns, shelter-in-place orders, limiting public gatherings, closing entertainment venues, self-quarantining at home, and social distancing, resulting greater levels of social isolation and difficulties in maintaining traditional social relationships. Therefore, loneliness has become one of the major public health concerns during the pandemic [2]. Evidence from empirical studies has shown that loneliness is an important risk factor for a myriad of deleterious physical and mental consequences, including early mortality, cardiovascular diseases, metabolic syndrome, suicidal behaviors, depressive symptoms, and cognitive decline [3–9]. Similarly, during this pandemic, population-based studies have demonstrated significant associations of feelings of loneliness with increased risk of depression, anxiety, psychological distress, poor sleep, alcohol and drug use, and poor quality of life [10–14]. Importantly, researchers found that, compared to adults who were not lonely, lonely adults were significantly less likely to engage in a variety of individual COVID-19 preventive behaviors [15]. Therefore, a greater understanding on loneliness in the context of COVID-19 pandemic may not only be helpful for the development of measures to improve people’s health and mental wellbeing but also facilitate efforts in combating the spread of COVID-19.

Several studies have examined loneliness among the general population affected by the pandemic. However, debates exist on whether there is an increase in the level of loneliness of the general population. For example, in United States, a longitudinal study of a nationwide adult population found no significant mean-level changes in loneliness before and after the outbreak of COVID-19, while in United Kingdom, results of comparison between pre- and peri-pandemic showed a significant increase in level of loneliness after the onset of COVID-19 pandemic in the adult population [16, 17]. The prevalence of loneliness in the general population also varies considerably, for example, during the COVID-19 pandemic, reported rates of loneliness in the general public in United Kingdom ranged from 27.0 to 50.8 % [10, 17, 18]. Factors identified to be associated with loneliness during the pandemic are consistent with those during the non-pandemic era: female gender, young age, living alone, inadequate social support, and major medical conditions [10, 16–20]. Nevertheless, because existing studies seldom considered pandemic characteristics as potential correlates of loneliness (i.e., risk perception of COVID-19), the relationship between loneliness and pandemic-specific factors remains poorly understood [10]. For example, only one previous study reported the significant association of loneliness with more time exposed to news about COVID-19 [13].

Most of the above-reviewed studies are conducted in western countries. In mainland China, to the best of our knowledge, only one study has assessed loneliness in Chinese population and the instrument used was the UCLA loneliness scale [21]. Despite the 74.6 % prevalence of severe loneliness among the Chinese public, the overall level of loneliness decreased, because in this study 25.4 % reported decreased level of loneliness compared to the pre-pandemic era while 17.4 % reported increased level of loneliness. The study found that reduced interactions with others was significantly associated with increased level of loneliness, which is also a commonly reported risk factor for loneliness [22]. Due to the small sample size of this study (*n* = 138), the generalizability of its findings is very limited.

The pandemic also disrupted mental health services in most countries despite a high demand for mental health services [23]. In China, the COVID-19 epidemic has resulted in unprecedented levels of mental health needs but most of the persons in need receive no mental health care [24]. Understanding factors associated with mental health services utilization is necessary, which may inform targeted care delivery approaches in the context of COVID-19 pandemic. During the non-pandemic era, loneliness is a significant predictor of greater numbers of physician visits and hospitalizations, increased contacts with community nurses, and increased use of outpatient care in the elderly population [25–28]. In addition, loneliness is associated with mental health problems, which in turn are associated with utilization of mental health services [29]. Because of these, we speculated that loneliness was associated with use of mental health services among the general population during the pandemic. However, an unknown question is that to what extent the loneliness-utilization relationship could be explained by mental health problems. Given that perceiving a need for mental health care is a strong driver of use of mental health services, we also speculated that loneliness was associated with perceived needs for mental health care [30]. Unfortunately, few studies have focused on the relationships between loneliness and mental health needs and services use in the context of COVID-19 pandemic.

Considering the above knowledge gaps, the present study examined prevalence and correlates of loneliness in the general population during the COVID-19 outbreak in China, as well as its association with mental health needs and services utilization.

## Methods

### Participants

Participants were 7741 adults from a nationwide cross-sectional survey, which was carried out from January 27 to February 2, 2020, the peak period of COVID-19 outbreak in China [24]. Because of concerns of COVID-19 infection and the urgent need for mental health data during the crisis period, participants were recruited via social media platforms and popular media outlets in Wuhan, the epicenter of COVID-19 in China. Chinese people aged 16 years or older, were willing to participate, and were not COVID-19 patients were eligible for this study.

The survey protocol was approved by the Ethics Committee of Wuhan Mental Health Center. All participants signed informed consent online.

### Procedures and measures

All subjects completed the self-administered questionnaire online. Socio-demographic variables collected were age, gender, marital status, education, and employment status. According to places where participants were located at the time of the survey and during the previous two months prior to the survey, they were categorized into four mutually exclusive groups: Wuhan residents (those currently located in Wuhan), migrants from Wuhan (those previously located in Wuhan and currently located in parts of China other than Wuhan), other Hubei residents (those who currently located in other parts of Hubei and not previously in Wuhan), and residents of other provinces of China (those currently located in other provinces of China and not previously in Wuhan).

Characteristics of the COVID-19 epidemic. Four questions were used: “How severe do you feel the COVID-19 epidemic is in the city/county of your current residence?” (severe vs. not severe), “Do you have any family member or close relative infected with severe acute respiratory syndrome coronavirus 2 (SARS-CoV-2)?” (yes vs. no), “Do you have any colleague, friend or classmate infected with SARS-CoV-2?” (yes vs. no), and “Do you have any neighbor of your community/village infected with SARS-CoV-2?” (yes vs. no).

Common mental health problems. The validated 12-item Chinese General Health Questionnaire (GHQ-12) was used to screen for common mental health problems [31]. Total score of GHQ-12 ranges between zero and 12 and a cut-off score of three or greater is considered for the presence of common mental health problems in China [32].

Feelings of loneliness. A single-item self-report question was used to ask participants how often they feel lonely in recent days [22]. The question was assessed on a five-point Likert scale: 1 = never, 2 = seldom, 3 = sometimes, 4 = often, 5 = always. This single-item measure of loneliness is widely used in previous studies [33–35]. In accordance with previous studies, respondents answering “sometimes”, “often”, and “always” were classified as having the feelings of loneliness [36, 37].

Mental health needs and services utilization. The two questions used were “During recent days, did you recognize that you need professional help from mental health specialists because of your high level of stress, bad emotions, poor sleep, or other mental health problems? These specialists include psychiatrists, psycho-therapists, and psychological counselors” and “During recent days, did you seek any help from mental health specialists for your mental health problems?”.

### Statistical analysis

Prevalence rates of loneliness in the whole sample and different subgroups were calculated. Chi-square test was used to compare rates between subgroups according to socio-demographic and epidemic characteristics. Multiple logistic regression with a backward stepwise entry of all significant variables in univariate analysis was used to identify factors associated with loneliness. We used Chi-square test to compare rates of mental health needs between lonely and not lonely residents. The independent association of loneliness with mental health needs was further examined with multiple logistic regression analysis which included perceived needs for mental health care the outcome variable, loneliness as the predictor, and socio-demographic and epidemic characteristics and common mental health problems all together as covariates. The association between loneliness and mental health services utilization was examined in the same manner. Odds ratios (ORs) and their 95 % confidence intervals (95 %CIs) were used to quantify associations between factors and the outcome variables. The statistical significance level was set at P < 0.05 (two-sided). SPSS software version 15.0 package (SPSS Inc., Chicago, IL, USA) was used for all analyses.

## Results

The final sample consisted of 2539 men (32.8 %) and 5202 women (67.2 %) and the mean age was 33.1 years (range: 16–87). Detailed socio-demographic and epidemic characteristics were displayed in Table 1.

**Table 1:** Sample characteristics, prevalence rates of loneliness by socio-demographic and COVID-19 epidemic characteristics, and results of multiple logistic regression analysis on factors associated with loneliness

Characteristics		Total sample (*n* = 7741), N (%, percentage among the total sample)	Lonely residents (*n* = 1874), N (%, prevalence of loneliness)	χ2	P	OR (95% CI)	P
Subpopulation	Wuhan residents	2617 (33.8)	662 (25.3)				
	Migrants from Wuhan	930 (12.0)	253 (27.2)				
	Other Hubei residents	633 (8.2)	141 (22.3)				
	Residents of other provinces in China	3561 (46.0)	818 (23.0)	10.498	0.015		
Gender	Male	2539 (32.8)	633 (24.9)				
	Female	5202 (67.2)	1241 (23.9)	0.074	0.300		
Age-group (years)	16–29	3166 (40.9)	889 (28.1)			1.36 (1.05, 1.76)	0.020
	30–49	3985 (51.5)	875 (22.0)			1.19 (0.95, 1.48)	0.133
	50+	590 (7.6)	110 (18.6)	46.820	< 0.001	1	
Marital status	Never-married	2975 (38.4)	862 (29.0)			1.47 (1.26, 1.73)	< 0.001
	Others^a^	347 (4.5)	106 (30.5)			1.72 (1.35, 2.19)	< 0.001
	Married	4419 (57.1)	906 (20.5)	77.515	< 0.001	1	
Employment status	Employed	5846 (75.5)	1360 (23.3)				
	Unemployed	476 (6.1)	116 (24.4)				
	Students	1419 (18.3)	398 (28.0)	14.251	0.001		
Education	Middle school and below	1269 (16.4)	313 (24.7)				
	Associate’s degree and above	6472 (83.6)	1561 (24.1)	0.172	0.678		
Perceived severity of the epidemic in current residence place	Severe	2548 (32.9)	645 (25.3)				
	Not severe	5193 (67.1)	1229 (23.7)	2.529	0.112		
Having family members or close relatives infected with SARS-CoV-2^b^	Yes	94 (1.2)	34 (36.2)			1.64 (1.06, 2.53)	0.026
	No	7647 (98.8)	1840 (24.1)	7.420	0.006	1	
Having colleagues, friends or classmates infected with SARS-CoV-2^b^	Yes	736 (9.5)	229 (31.1)			1.62 (1.36, 1.92)	
	No	7005 (90.5)	1645 (23.5)	21.137	< 0.001	1	< 0.001
Having infected neighbors of the same community/village	Yes	829 (10.7)	221 (26.7)				
	No	6912 (89.3)	1653 (23.9)	3.037	0.081		

^a^“Others” included re-married, co-habiting, separated, divorced, and widowed

^b^*SARS-CoV-2*: Severe Acute Respiratory Syndrome Coronavirus 2

A total of 1874 participants felt lonely in recent days and the corresponding prevalence of loneliness was 24.2 %. Results of univariate analysis showed that rates of loneliness were more likely to be higher in migrants from Wuhan, participants aged 16–29 years, persons with a marital status other than “married”, students, residents having infected family members or close relatives, and individuals having infected colleagues, friends, or classmates (*P* ≤ 0.015) (Table 1). Multiple logistic regression analysis revealed that young age (16–29 years) (OR = 1.36, *P* = 0.020), marital status of never-married (OR = 1.47, *P* < 0.001), marital status of “others” (re-married, co-habiting, separated, divorced, and widowed) (OR = 1.72, *P* < 0.001), having infected family members or close relatives (OR = 1.64, *P* = 0.026), and having infected colleagues, friends, or classmates (OR = 1.62, *P* < 0.001) were significantly associated with loneliness (Table 1).

Rates of mental health needs (17.4 % vs. 4.9 %, *P* < 0.001) and services utilization (2.7 % vs. 1.0 %, *P* < 0.001) were significantly higher in lonely than not lonely participants. Despite this, among the 327 lonely persons with perceived needs for mental health care, only 50 (15.3 %) sought help from mental health professionals. After adjusting for socio-demographic and epidemic characteristics and common mental health problems, loneliness was still significantly associated with mental health needs (OR = 2.50, *P* < 0.001) and services utilization (OR = 1.62, *P* = 0.020) (Table 2).

**Table 2:** Multiple logistic regression analyses on associations of loneliness with perceived needs for mental health care and mental health service use among Chinese residents during the COVID-19 epidemic, adjusting for the confounding effects of socio-demographic and epidemic characteristics and common mental health problems

Variables		Perceived needs for mental health care		Use of mental health services	
		OR (95 %CI)	P	OR (95% CI)	P
Loneliness	Yes	2.50 (2.08, 3.00)	< 0.001	1.62 (1.08, 2.44)	0.020
	No	1		1	
Subpopulation	Wuhan residents	0.99 (0.76, 1.29)	0.958	1.32 (0.74, 2.34)	0.352
	Migrants from Wuhan	1.00 (0.75, 1.34)	0.998	1.19 (0.65, 2.20)	0.571
	Other Hubei residents	0.88 (0.60, 1.29)	0.515	1.03 (0.44, 2.38)	0.955
	Other residents	1		1	
Gender	Female	1.38 (1.12, 1.69)	0.002	0.76 (0.51, 1.14)	0.186
	Male	1		1	
Age-group (years)	16–29	1.30 (0.82, 2.05)	0.263	0.37 (0.13, 1.05)	0.063
	30–49	1.26 (0.85, 1.87)	0.254	0.80 (0.35, 1.83)	0.590
	50+	1		1	
Marital status	Others^a^	1.84 (1.30, 2.60)	0.001	2.22 (1.20, 4.13)	0.011
	Never-married	0.95 (0.72, 1.25)	0.700	2.42 (1.19, 4.91)	0.014
	Married	1		1	
Employment status	Students	1.10 (0.77, 1.58)	0.609	1.99 (1.07, 3.71)	0.031
	Unemployed	0.94 (0.70, 1.28)	0.709	0.40 (0.12, 1.33)	0.134
	Employed	1		1	
Education	Associate’s degree and above	0.98 (0.75, 1.29)	0.893	0.86 (0.49, 1.50)	0.591
	Middle school and below	1		1	
Perceived severity of the epidemic in current residence place	Severe	0.99 (0.79, 1.25)	0.929	0.77 (0.46, 1.27)	0.298
	Not severe	1		1	
Having family members or close relatives infected with SARS-CoV-2^b^	Yes	2.33 (1.03, 5.26)	0.042	0.81 (0.19, 3.48)	0.779
	No	1		1	
Having colleagues, friends or classmates infected with SARS-CoV-2^b^	Yes	1.33 (1.02, 1.73)	0.036	1.86 (1.07, 3.22)	0.027
	No	1		1	
Having infected neighbors living in the same community/village	Yes	1.22 (0.94, 1.57)	0.132	1.08 (0.62, 1.89)	0.788
	No	1		1	
Common mental health problems	Yes	6.09 (4.96, 7.48)	< 0.001	4.34 (2.77, 6.80)	< 0.001
	No	1		1	

^a^“Others” included re-married, co-habiting, separated, divorced, and widowed

^b^SARS-CoV-2: Severe Acute Respiratory Syndrome Coronavirus 2

## Discussion

As far as we know, this is the first large-scale study in China that examined the prevalence and correlates of loneliness in the general population during the COVID-19 epidemic, as well as its associations with mental health needs and services utilization. Because the COVID-19 epidemic has been associated with increased level of mental health needs in affected people but the available mental health services resources are insufficient to meet their needs [24], our findings on relationships between loneliness and mental health needs and services utilization are potentially useful for the planning and development of appropriate mental health services during the COVID-19 epidemic.

The main finding of this study is the 24.2 % prevalence of loneliness among Chinese residents during the COVID-19 epidemic. Compared to studies using similar definitions of loneliness, this prevalence estimate is higher than those reported in other cohorts during the non-pandemic era, including the 18.3 % prevalence of “often feel lonely” in Chinese migrant workers [38], the 22.1 % prevalence of “feel lonely at least sometimes” in Chinese university fresh students [39], the 15.6 % prevalence of “feel lonely” in Chinese older adults [40], the 20.9 % prevalence of “feel lonely often or sometimes” in Norwegian adults [34], the 18.4 % prevalence of “feel lonely at least rarely” in adolescents in Ghana [41], the 18.6 % prevalence of “feel lonely at least sometimes” in general population in Indonesia [42], and the 17.9 % prevalence of “feel lonely often or occasionally” in older patients from general practices in Denmark [43]. The higher prevalence rate of loneliness in our sample in comparison to those in older adults and young adults (i.e., adolescent and university students) from previous studies are worth noting because the two subpopulations are generally believed to be at higher risk for loneliness than other age-groups [34, 42], suggesting the elevated risk of loneliness of Chinese general population during the COVID-19 epidemic.

In multiple logistic regression analysis, we found significant and independent associations of loneliness with young age and marital status of never-married and “others” in COVID-19 affected Chinese residents. Similar associations have been reported in population-based studies during both pandemic and non-pandemic periods [10, 44–46]. Due to the physical distancing requirement, Chinese young adults more rely on social media and mobile devices to keep connections with friends and others. However, young adults with high use of social media are more likely to feel lonely because of the lack of intimacy of real-life face-to-face human interaction [47]. This may explain the high prevalence of loneliness in young adults in our study. Because spouse or family support is of particular importance for coping with the stress caused by the COVID-19 pandemic, the association between loneliness and marital status of “other than married” is expected. Since participants who have family members and friends infected by COVID-19 are more likely to be isolated and placed on quarantine, associations of loneliness with having infected family members or close relatives and having infected colleagues, friends, or classmates are also expected.

Loneliness is “the unpleasant experience that occurs when a person’s network of social relations is deficient in some important way, either quantitatively or qualitatively” [48]. Accordingly, in non-pandemic era, commonly reported factors associated with loneliness are marital status of “unmarried”, living alone, social exclusion, less frequent contact with neighbors, and poor family and non-family relationships [22, 49, 50]. However, in the present study, epidemic-related factors such as having SARS-CoV-2-infected acquaintances were associated with loneliness. This finding suggests the negative impact of macro environment on the mental wellbeing of Chinese residents during the pandemic era; for example, in the context of traditional Chinese culture, people prefer face-to-face social interactions to maintain meaningful interpersonal relationships, but this social connection was interrupted by mass quarantine and social distancing requirements, resulting in feelings of loneliness.

In this study, lonely individuals were 2.5-fold more likely to have mental health needs and 1.6-fold more likely to seek treatment from mental health professionals. Researchers have speculated that the special clinician-patient relationship may play a social role for lonely persons who need someone to talk to [26]. In addition, the poor mental health of lonely individuals may also explain this phenomenon [51]. However, results from further adjustment analyses showed significant associations of loneliness with mental health needs and services utilization, which were independent from socio-demographic and epidemic characteristics and common mental health problems. This suggests that loneliness may be an important determinant of mental health needs and services utilization in Chinese general population affected by the COVID-19 epidemic. Despite more mental health needs and greater utilization of mental health services in lonely persons, we found that 84.7 % of the lonely persons with mental health needs did not seek mental health help. Unlike western countries, community-based social work services are still at their very early stage in China [52]. This high level of unmet mental health needs may be attributed to the limited access to and provision of psychosocial services during the COVID-19 outbreak, including social work services.

Findings from the present study need to be interpreted with caution due to several limitations. First, this study was conducted online and, as shown in Table 1, the recruited sample was overrepresented by women and young adults and socioeconomically advantaged persons, so the sample representativeness is limited. There is evidence that persons of a high socioeconomic status is less likely to feel lonely [53]. Second, due to stigma associated with loneliness, the single-item measure of loneliness in our study has been criticized for underestimating the actual prevalence of loneliness [54]. As a result of these, our study may underestimate the true prevalence of loneliness in the Chinese population affected by the pandemic. Third, because this is a cross-sectional study, causal relationships between identified correlates and loneliness and mental health needs and services utilization can not be ascertained. Fourth, some other potential risk factors of loneliness such as lack of social support and social isolation were not measured in this study. Longitudinal studies are warranted to determine causal relationships between loneliness and mental health needs and services utilization.

## Conclusions

In summary, during the COVID-19 epidemic, feelings of loneliness are common among Chinese residents and the presence of loneliness is associated with high levels of mental health needs and greater services utilization. China is a mental health services resource-poor country and, like other countries in the world, its mental health services were also disrupted during the outbreak [24], so the limited mental health services resources became more inadequate to meet Chinese residents’ mental health needs during the epidemic. Given many negative health outcomes associated with loneliness, reducing or preventing loneliness is potentially beneficial for improving the mental and physical wellbeing of Chinese residents during the epidemic. Importantly, our findings suggest that mitigating loneliness may reduce people’s use of the limited mental health services resources during the pandemic, potentially ensuring those who are most in need of mental health services (i.e., individuals at high risk of suicide and experiencing a psychotic episode) receive timely and necessary treatment. So there is an urgent need for health authorities and health workers to address the epidemic of loneliness in COVID-19 affected population. Efforts to alleviate loneliness in Chinese residents may be useful to target on those who are young, have a marital status of “other than married”, have infected family members or close relatives, and have infected colleagues, friends, or classmates. Services for COVID-19 affected residents should include periodic evaluation of psychosocial problems and expanded social supports that specifically focus on improving their mental wellbeing.

## Data Availability

All relevant data are available without any restriction. Requests for data can be sent to Dr. Bao-Liang Zhong at haizhilan@gmail.com.

## Abbreviations

COVID-19: Coronavirus Disease 2019
OR: Odds ratio
UCLA: University of California, Los Angeles
SARS-CoV-2: Severe acute respiratory syndrome coronavirus 2
SPSS: Statistical Package for the Social Sciences
GHQ-12: 12-item General Health Questionnaire
